# Extreme Oncoplasty: Oncologically Safe When Compared with Mastectomy

**DOI:** 10.1245/s10434-024-15791-y

**Published:** 2024-07-13

**Authors:** Deena Hossino, Melvin J. Silverstein, Nirav Savalia, Margaret Jayich, Sadia Khan

**Affiliations:** 1 Hoag Memorial Hospital Presbyterian, Newport Beach, CA USA; 2 Keck School of Medicine, University of Southern California, Los Angeles, CA USA

**Keywords:** Avoiding mastectomy, Extreme oncoplasty, Multifocal/multicentric breast tumors, Oncoplastic breast conservation

## Abstract

**Background:**

Extreme oncoplasty is a breast-conserving operation using oncoplastic techniques in a patient who does not meet the traditional criteria for breast conservation and in whom most physicians would suggest a mastectomy. These tumors are generally multicentric and/or multifocal, they span more than 50 mm, or they can be large recurrences in a previously irradiated breast.

**Methods:**

A prospective single institution database was queried from 2008 through mid 2023 for patients who met the criteria for extreme oncoplasty and were treated with excision plus whole-breast radiation therapy (WBRT) or mastectomy without WBRT. Patients with recurrent breast cancer were excluded. Endpoints were local, regional, and distant recurrence as well as overall and breast-cancer-specific survival.

**Results:**

272 patients were treated with oncoplastic mammaplasty, using a standard or split reduction excision followed by postoperative WBRT. An additional 101 patients elected to be treated with mastectomy without postoperative radiation therapy. With a median follow-up of 7 years, there were no significant differences in local, regional, or distant recurrence, nor in breast-cancer-specific survival or overall survival.

**Conclusions:**

We strongly support extreme oncoplasty plus WBRT as the default procedure of choice for patients with large multifocal/multicentric lesions amenable to reconstruction with volume displacement mammaplasty.

## Introduction

In 1990, a National Institutes of Health (NIH) Consensus Development Conference concluded that “breast conservation treatment is an appropriate method for the majority of women with stage I and II breast cancer and is preferable because it provides survival equal to total mastectomy and axillary dissection while preserving the breast.”^1,2^ However, multicentric tumors and cancers greater than 5 cm were excluded. More recently, National Comprehensive Cancer Network (NCCN) guidelines have included lesions larger than 5 cm as candidates for breast conservation. Multicentricity, too, is no longer an absolute contraindication to breast conservation, on the basis of the results of ACOSOG Z11102.^3^

Standard-breast conservation techniques may be difficult for tumors spanning more than 50 mm as a wide excision creates a large cavity, often leading to a difficult closure and deformed breast contour. Oncoplastic breast-conservation surgery has been proposed as a solution to this problem. Oncoplastic breast surgery is defined by the American Society of Breast Surgeons (ASBrS) as “breast conservation surgery incorporating an oncologic partial mastectomy with ipsilateral defect repair using volume displacement or volume replacement techniques with contralateral symmetry surgery as appropriate.” This allows for correction of the defect using volume displacement or volume replacement techniques. During the last 10 years, there has been an increasing popularity of use of oncoplastic breast surgery.^4^

Extreme oncoplasty, as defined by Silverstein et al., is a breast-conserving operation using oncoplastic techniques in a patient who does not meet the traditional criteria for breast conservation and in whom most physicians would suggest a mastectomy.^5,6^ These tumors span more than 50 mm, are generally multifocal and/or multicentric, or they can be large recurrences in a previously irradiated breast. The term “extreme oncoplasty” was officially coined in 2015, however the procedure was used beginning in 2008 and initially called radical conservation.^5,6^ This paper reviews long-term data on local, regional, and distant recurrence, as well as breast-cancer-specific survival and overall survival, between patients undergoing extreme oncoplasty followed by whole-breast radiation therapy (WBRT) versus a similar cohort undergoing mastectomy without WBRT accrued during the same time period.

## Methods

Tumor size was defined as the diameter of the largest invasive component of a tumor complex. If the tumor was pure ductal carcinoma in situ (DCIS), then its largest diameter was recorded as its size. For this study, tumor span was of much greater importance. Tumor span was defined as the largest distance encompassing the entire tumor complex. It included all multifocal or multicentric components and all DCIS. Tumor span was the primary measurement used in this study and was determined by the pathologist, using serial sectioning with the aid of imaging studies.

For example, a single (not multifocal or multicentric) DCIS or invasive tumor might span 75 mm. If so, its size and span were the same and recorded as 75 mm. However, if a tumor had a 7-mm invasive component within a 75-mm area of DCIS, the size was recorded as 7 mm (T1b) while the span was recorded as 75 mm. If there were two areas of invasive cancer, one measuring 2 cm and a second separate area measuring 3 cm, and they were separated by 2 cm, this tumor was recorded as size 3 cm (T2, size of the largest invasive component) while the span (the largest distance including all components) was recorded as 7 cm. Tumor size was used for tumor–node–metastasis (TNM) staging.

A prospective institutional review board (IRB)-approved database was queried for patients with a new primary breast cancer, treated at a single institution, from 2008 through mid-2023, who met the criteria for extreme oncoplasty and were offered that procedure. Patients with recurrent cancer were not included in this study. A total of 373 patients with tumors that spanned more than 50 mm that may have been multifocal or multicentric met the criteria; 101 patients declined extreme oncoplastic surgery and elected to undergo a mastectomy, while 272 patients elected to be treated with extreme oncoplastic surgery followed by postoperative WBRT. All patients had bilateral mammograms, ultrasound of the involved breast and axilla, and bilateral magnetic resonance imaging (MRI). Race and ethnicity were not collected in this database.

Endpoints were local, regional, and distant recurrence as well as breast-cancer-specific survival and overall survival. Kaplan–Meier analyses were used to predict recurrence and survival probabilities. Curves were compared with the log-rank test. Independent variables were compared with chi-squared and means between groups with the *t*-test.

## Results

Patient characteristics are compared between the two groups in Table 1. The average tumor span was similar for both groups: 74 mm for extreme oncoplasty and 77 mm for mastectomy. Although the tumor span was similar in both groups, the T category and tumor stage varied widely between the two groups (Table 2). In both groups, the patients were more likely to be estrogen receptor (ER) positive, progesterone receptor (PR) positive, and human epidermal growth factor receptor 2 (HER2) negative with an average nuclear grade of 2.40 and average Ki67 of 25%. A total of 187 patients (50%) had multifocal tumors, while 54 (14.5%) had multicentric tumors, involving more than one breast quadrant.

**Table 1:** Patient characteristics

	Extreme + WBRT	Mastectomy no RT	*p*-value
Percent invasive	228/272 (84%)	59/101 (58%)	< 0.001
Average age	57 years	52 years	< 0.001
Average size	74 mm	77 mm	NS (*p* = 0.28)
Average nuclear grade	2.40	2.40	NS (*p* =0.99)
Whole-breast RT	Yes	No	NA
ER positive	232/272 (85%)	80/101 (79%)	NS (*p* = 0.15)
PR positive	198/272 (73%)	72/101 (71%)	NS (*p* = 0.77)
HER2 positive (invasive only)	48/228 (21%)	12/55 (22%)	NS (*p* = 0.31)
Average Ki67 (invasive only)	24.7%	25.0%	NS (*p* = 0.64)
Node positive	82/255 (32%)	15/97 (15.4%)	*p* = 0.002
Node positive (invasive only)	81/221 (36.6%)	15/57 (26.3%)	NS (*p* = 0.14)

*WBRT* whole-breast radiation therapy, *RT* radiation therapy, *NS* nonsignificant

**Table 2:** T category and TMN staging

	Extreme + WBRT	Mastectomy no RT	*p*-value
T category			
Tis	41 (15.1%)	42 (41.6%)	< 0.001
T1	81 (29.8%)	25 (24.8%)	0.338
T2	67 (24.6%)	11 (10.9%)	0.004
T3	76 (27.9%)	23 (22.8%)	0.032
T4	7 (2.6%)	0%	0.232
TNM stage			
Stage 0	41 (15.1%)	42 (41.6%)	< 0.001
Stage 1	69 (25.4%)	21 (20.8%)	0.359
Stage 2	100 (36.8%)	26 (25%)	0.045
Stage 3	57 (21.0%)	11 (10.9%)	0.025
Stage 4	5 (1.8%)	1 (1%)	0.563

*WBRT* whole-breast radiation therapy, *RT* radiation therapy

The groups differed statistically in three factors: extreme oncoplasty patients were more likely to have invasive disease (84% versus 58%; *p* < 0.001), they were older (57 versus 52 years; *p* < 0.001), and they were more likely to be node positive (32% versus 15.4%; *p* = 0.002).

**Fig. 1:**
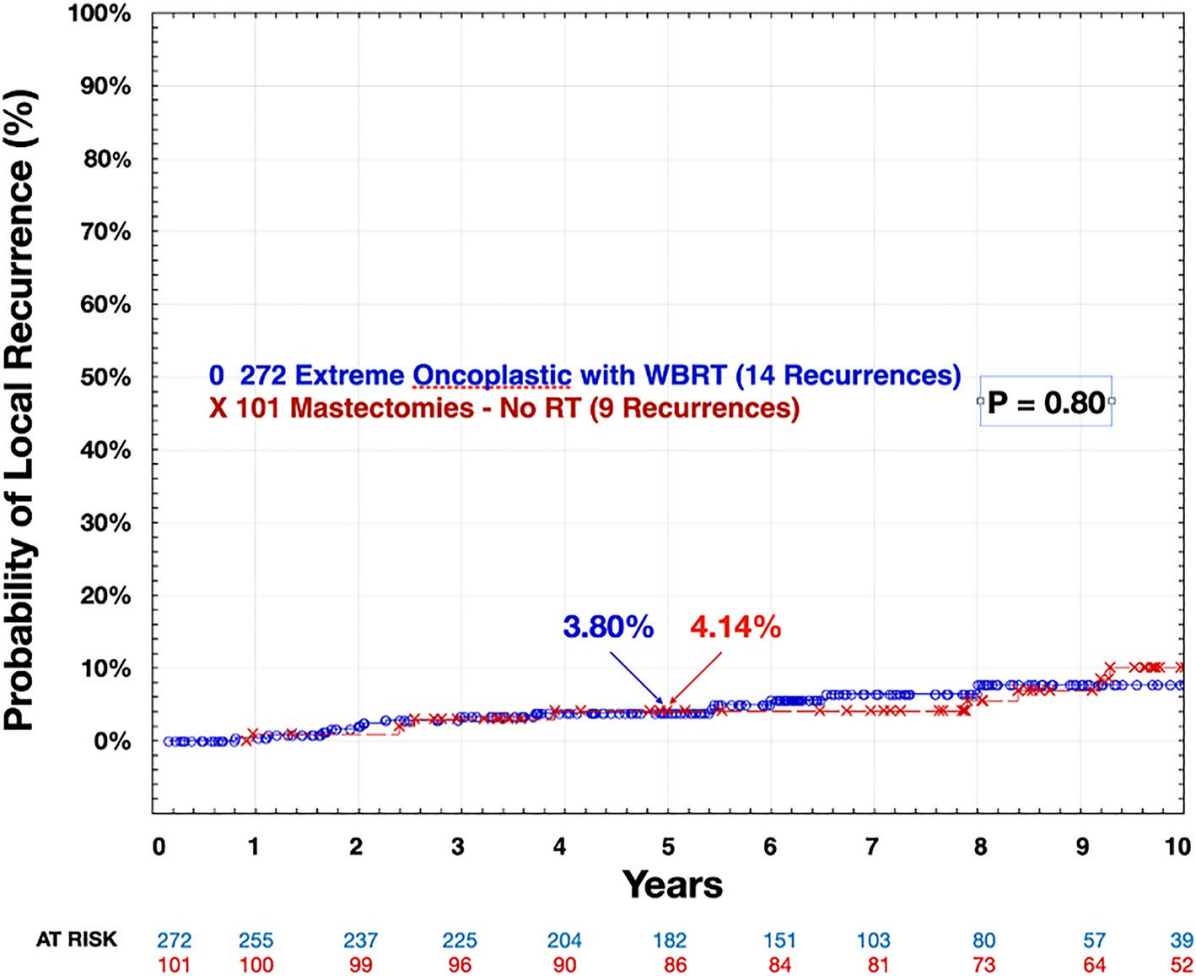
Extreme oncoplasty with WBRT versus mastectomy with no RT local recurrence*. WBRT* whole-breast radiation therapy, *RT* radiation therapy

With a median follow-up of 5 years, there were no significant differences in local, regional, or distant recurrence, nor in breast-cancer-specific survival or overall survival. Among the 272 patients treated with extreme oncoplasty and WBRT, there were 14 local recurrences and 9 deaths, 5 of which were breast cancer related. Among the 101 patients treated with mastectomy, there were 9 local recurrences and 3 deaths, 2 of which were breast cancer related. The predicted local recurrence rate at 5 years for the extreme oncoplastic group was 3.80%; for the mastectomy group, it was 4.14% (*p* = 0.80) (Fig. 1). The overall survival at 5 years was 98.0% for the extreme oncoplastic group and 98.2% for the mastectomy group (*p* = 0.35) (Table 3).

**Table 3:** Extreme Oncoplasty with WBRT vs Mastectomy with No RT

	Extreme + WBRT (%)	Mastectomy No RT (%)	*P* value
5-Yr Local Recurrence Probability	4.14	3.80	0.80
5-Yr Axillary Recurrence Probability	1.1	0.9	0.39
5-Yr Distant Recurrence Probability	2.1	4.6	0.28
5-Yr Overall Survival	98.0	98.2	0.35
5-Yr Breast Cancer Survival	99	98.2	0.50

WBRT= Whole Breast Radiation Therapy

RT= Radiation Therapy

## Discussion

Extreme oncoplasty allows patients with large multifocal/multicentric lesions to benefit from a more natural cosmetic appearance, like their counterparts with lesser disease, rather than undergo mastectomy. This study provides the longest follow-up data (median 7 years) and largest series of extreme oncoplasty patients to date. The results confirm that the procedure is safe, with no significant difference in recurrence, breast-cancer-specific survival, or overall survival. It shows that, in spite of large volumes of disease, extreme oncoplasty yields results similar to the standard treatment of mastectomy. For women who do not meet the traditional criteria for breast conservation, extreme oncoplasty may be an alternative. In addition to known improved quality of life and sensation, this study demonstrates that extreme oncoplasty is equivalent to mastectomy in terms of local recurrence, regional recurrence, distant recurrence, breast-cancer-specific survival, and overall survival.

The patient characteristics varied between the two groups (Tables 1 and 2). Interestingly, there were more patients with invasive disease who underwent extreme oncoplasty plus WBRT than those who underwent mastectomy alone. In spite of this, the extreme group fared just as well as the mastectomy group. There were more patients with positive lymph nodes in the extreme oncoplasty group, but when only patients with invasive disease were compared, the difference was no longer significant.

Although the tumor span was similar in both groups (74 mm for extreme oncoplasty and 77 mm for mastectomy), the T category and TMN stage varied widely between the groups (Table 2). This occurred because of the difference in the way size and span were calculated for this study. As noted above, a 2-mm invasive cancer within a 80-mm DCIS had a size of 2 mm and a span of 80 mm. It is clear that these two groups of patients are not easily compared using T category or TNM staging. Regardless of these differences, the outcomes for all measured endpoints were similar between the two groups.

All of the patients in the extreme oncoplasty group received postoperative radiation therapy. Irradiation may result in fibrosis and contracture of the breast that continues to manifest for years after the initial insult.^7^ When performing the initial oncoplastic surgery, we allow for approximately 20% shrinkage of the irradiated breast during the first 5–10 years post surgery. None of our mastectomy patients received radiation therapy, but had they, there would have been a significant risk of capsular contraction and other radiation-induced complications.

Extreme oncoplasty cases are typically completed in a single operation as an outpatient surgery. There is less postoperative pain than mastectomy, and there are usually no drains required. The reduced breast generally has good sensation, whereas the post-mastectomy breast generally has little or none.

With improved survivorship in breast cancer, greater emphasis has been placed on the post-treatment quality of life. Patient satisfaction studies have demonstrated that significantly larger portions of lumpectomy patients returned to their baseline breast satisfaction, psychosocial well-being, and physical well-being when compared with mastectomy patients.^8^ Breast-conserving surgery leads to better outcomes in terms of body image and future perspectives.^9^

However, it is expected that a significant portion of these lumpectomies will result in major deformities and asymmetries, which can have a negative impact on quality of life and can serve as a reminder of the disease. For women considering breast-conservation therapy, achieving a good esthetic outcome is one of the main goals. In a study looking at patient satisfaction in standard lumpectomy versus oncoplastic surgery, patients who underwent oncoplastic surgery achieved satisfactory results 84–89% of the time compared with the lumpectomy group at 60–80%.^10^ When comparing oncoplastic surgery versus mastectomy with autologous reconstruction, those who underwent oncoplastic surgery were found to have higher satisfaction when it came to breast appearance, overall satisfaction, and emotional and physical wellbeing.^11^

There are other quality-of-life considerations beyond appearance and the emotional impact. After a mastectomy, the patient loses sensation to the chest wall, which can have a devastating impact on self-image and self-esteem. Meanwhile, extreme oncoplasty, in spite of the removal of a large volume of disease, allows much or all sensation to be preserved.

While there are no prospective randomized survival data comparing mastectomy with breast conservation for large, greater than 5 cm, multifocal/multicentric breast cancer, studies suggest that survival is equivalent. Bleicher et al.^12^ used Surveillance, Epidemiology, and End Results (SEER) data to study 5685 patients aged ≥ 66 years with tumors greater than 5 cm. Breast-cancer-specific survival and overall survival were equivalent in both the mastectomy and breast-conservation groups. Breast conservation is clearly superior to mastectomy cosmetically and psychosocially. It may also be superior oncologically. A study of 132,149 patients with tumors 4 cm or smaller and three or fewer positive nodes revealed a 3% better breast-cancer-specific survival at 10 years for breast conservation^13^. Similar results were found in studies from Sweden^14^ and the Netherlands^15^.

This series was limited by being a nonrandomized, single-institution study without collection of race, ethnicity, basal metabolic index, and other personal data.

## Conclusions

Many patients with breast cancer who do not meet traditional criteria for breast conservation are offered mastectomy as the only surgical option. In many cases, this deforming, life-changing operation is unnecessary, is overtreatment, and offers no recurrence or survival benefit when compared with extreme oncoplasty plus WBRT. Extreme oncoplasty offers superior cosmetic results and equivalence of local recurrence, regional recurrence, distant recurrence, breast-cancer-specific survival, and overall survival for patients with multicentric/multifocal tumors larger than 50 mm, regardless of surgical management. We endorse extreme oncoplasty plus WBRT as the default procedure of choice for patients with large multifocal/multicentric lesions amenable to reconstruction with local tissue rearranging mammaplasty.
